# Soluble Markers of Immune Activation Differentially Normalize and Selectively Associate with Improvement in AST, ALT, Albumin, and Transient Elastography During IFN-Free HCV Therapy

**DOI:** 10.20411/pai.v3i1.242

**Published:** 2018-09-07

**Authors:** Lenche Kostadinova, Carey L. Shive, Elizabeth Zebrowski, Brianna Fuller, Kelsey Rife, Amy Hirsch, Anita Compan, Anita Moreland, Yngve Falck-Ytter, Daniel L. Popkin, Donald D. Anthony

**Affiliations:** 1 The Louis Stokes VA Medical Center, Cleveland, Ohio; 2 Department of Medicine, University Hospitals Medical Center, and the Center for AIDS Research, Case Western Reserve University, Cleveland, Ohio; 3 Department of Dermatology, University Hospitals Medical Center, Case Western Reserve University, Cleveland, Ohio

**Keywords:** hepatitis C, cellular immunity, T cell, monocyte, inflammation

## Abstract

**Background:**

During chronic hepatitis C virus (HCV) infection, Aspartate Aminotransferase (AST) and Alanine Aminotransferase (ALT) levels mark active liver inflammation and tissue damage, while albumin reflects synthetic liver function and nutritional status. Transient Elastography (TE) is a clinical measure of liver stiffness that facilitates evaluation of liver damage stage. While a portion of the TE score is attributable to liver fibrosis and relatively irreversible damage, another component of the TE score is attributable to liver inflammation or edema. Markers of inflammation during chronic HCV infection include soluble markers of immune activation, which are also associated with morbid outcome (including cardiovascular disease and liver-disease progression). Whether soluble markers of immune activation or changes in their level during HCV therapy relate to normalization of AST, ALT, Albumin, or TE score, is not clear.

**Methods:**

We evaluated soluble markers of immune activation (plasma sCD14, IL-6, sCD163, autotaxin [ATX], and Mac2BP) and TE score, and their relationship in 20 HCV-infected patients before, during, and after HCV-directed IFN-free direct-acting antiviral (DAA) therapy. We evaluated normalization of parameters and the relationship between each over a 6-month window.

**Results:**

Before therapy, serum AST levels positively correlated with plasma levels of sCD14, sCD163, and Mac2BP, while ALT levels positively correlated with Mac2BP. Serum albumin level negatively correlated with plasma IL-6 and ATX levels. IFN-free therapy uniformly resulted in sustained virological response at 12 and 24 weeks after therapy completion. After initiation of therapy AST and ALT normalized, while levels of ATX, Mac2BP, sCD163, and TE score partially normalized over 6 months. Additionally, change in AST level and APRI score correlated with change in sCD163, IL-6, and Mac2BP levels, and change in ALT correlated with change in IL-6 and Mac2BP levels. Improvement in TE score correlated with a decrease in the level of sCD14 at week 4, and almost statistically significant with decrease in sCD14 at weeks 20-24 after initiation of IFN-free HCV therapy.

**Conclusions:**

Soluble markers of immune activation normalize or partially normalize at different rates after initiation of curative HCV DAA therapy, and TE scores improve, with wide variability in the degree of absolute improvement in liver stiffness from patient to patient. Decline magnitude of sCD14 was associated with improvement in TE score, while magnitude of improvement in AST correlated with reduction in sCD163 levels. These data provide support for a model where monocyte/Kupffer cell activation may account for a portion of the liver inflammation and edema, which is at least partially reversible following initiation of HCV DAA therapy.

## INTRODUCTION

Chronic hepatitis C infection is the most common cause of cirrhosis, end-stage liver disease, and hepatocellular carcinoma (HCC) in the United States [1, 2]. The yearly incidence of HCC in patients with untreated cirrhosis is 3%-7% [3]. Previously, pegylated interferon- and ribavirin-based therapies were effective in less than 50% of patients, while newer direct-acting antivirals (DAA) achieve sustained virological response (SVR) in over 90% of treated patients [4–6]. SVR leads to an overall reduction in all-cause mortality of 50-69%, and 74% in patients with cirrhosis [7, 8]. Patients who achieve SVR have a significantly lower incidence of HCC compared to those without SVR, but the absolute risk for HCC is still high in those with cirrhosis despite SVR [9]. Since patients with HCV associated cirrhosis who achieve SVR with IFN-free therapy remain at high risk for HCC, current standard practice is to regularly screen these patients for HCC using ultrasound, MRI, or CT scan imaging. Long-term management strategies will benefit from a clearer understanding of factors involved in, or predictive of, long-term morbidity in this patient population.

Transient elastography (TE) is an ultrasound-based method used to identify patients with liver fibrosis [10]. It has 91% specificity and an 87% sensitivity for detecting cirrhosis [11]. A recent meta-analysis indicated that there is a greater reduction of the TE score after DAA therapy compared to IFN-based treatment, with some data indicating a 50% decline in the TE score 6 months after DAA therapy, followed by a slower decline over the subsequent 5 years [12–15]. It is thought that liver stiffness during chronic infection is a combination of hepatic inflammation and fibrosis, while after viral eradication the component of inflammation abates, followed by a slower phase of fibrosis regression. In order to better understand this process more information is needed regarding predictors, and thus potential mechanistic targets, of liver health normalization after IFN-free HCV therapy.

Sustained inflammation and related fibrogenesis represent the basis of liver damage during chronic HCV infection [16–19]. The inflammatory milieu is characterized by a network of inflammatory chemokines and mediators of innate and adaptive immune system activation. There have been a number of soluble inflammatory markers, including sCD14, IL-6, sCD163, Mac2BP, and autotaxin (ATX), that have been identified to associate with liver fibrosis and elevated transaminase levels. We and others have identified the soluble inflammatory biomarkers sCD14, IL-6, and sCD163 as correlated with virological, hepatic, or functional immunological outcomes [20–22]. CD163 is a marker of activated macrophages. Increased levels of CD163 on the surface of macrophages in the liver have been observed in patients with hepatitis, and this is associated with increased levels of the shed form of sCD163 in serum [21, 23–25]. CD14 is a myeloid differentiation marker found on monocytes and macrophages [26, 27]. It is also a co-receptor for lipopolysaccha-ride (LPS), and elevated levels of serum or plasma sCD14 are thought to be reflective of monocyte activation [28]. We and others have also described autotaxin (ATX) to be elevated during HCV and HCV-HIV infection, and to be associated with the liver damage state [22, 29]. These markers are also associated with liver-disease progression [20, 30]. In our previous data set we showed that during IFN-free DAA HCV therapy there is normalization of the levels of ATX and sCD14 and partial normalization of sCD163 and Mac2BP [22]. Whether soluble markers of immune activation or changes in their level during HCV therapy relate to normalization of Aspartate Amino-transferase (AST), Alanine Aminotransferase (ALT), Albumin, or TE scores, has not been clear. In this data set we evaluated the normalization of parameters of immune activation, the relationship between each, and liver stiffness over a 6-month window directly after initiation of antiviral IFN-free DAA therapy.

## METHODS

### Study Participants

Study participants provided written informed consent for phlebotomy under protocols approved by the institutional review board for human studies at the Cleveland Veterans Affairs Medical Center. Twenty participants chronically infected with HCV, undergoing standard of care HCV DAA therapy, and who had received standard of care pre-therapy TE, were enrolled. Those who had follow-up TE (either as a standard of care or who agreed to a follow-up scan for the research protocol) at 6 months post therapy are the focus of the present analysis. Participants with chronic HCV infection were HCV antibody (seropositive for at least 6 months) and RNA positive (limit of detection 15 IU/ml) and underwent IFN-free therapy (sofosbuvir/ledipasvir+ribavirin (n = 9), sofosbuvir/ledipasvir (n = 7) or dasabuvir/ombitasvir/paritaprevir/ritonavir+ribavirin (n = 4)), for 8-12 weeks as part of standard care (Table 1).

**Table 1:** Study Participant Clinical Characteristics

Variable	Uninfected Donors	HCV pre-therapy	*P* value
Number	16	20	
Age (years)	57 (53-65)	64 (60,65)	
Race			
Caucasian	4 (25%)	3 (15%)	
African American	12 (75%)	17 (85%)	
Sex/Male	16 (100%)	20 (100%)	
HCV (IU/mL)		1,554,075 (583,048; 2,783,377)	
HCV genotype			
1		20 (100 %)	
AST (IU/mL)	23 (22, 24)	41 (29, 62)	0.003
ALT (IU/mL)	23 (21, 36)	53 (42,99)	0.004
PLT (x 10^3^/mm^3^)	214 (167, 249)	190 (169, 230)	
Albumin (g/dL)	4.15 (3.97, 4.2)	3.9 (3.6, 4.2)	
APRI score	0.22 (0.18, 0.32)	0.5 (0.35, 0.96)	0.003
Transient Elastography (kPa)		12.5 (10, 18)	

Values are presented as median (interquartile range) for each group

APRI: [(AST/45)/PLT] x 100

Transient elastography score normal range value is below 9.5

### Soluble Markers of Immune Activation

Blood was collected into K_2_EDTA tubes (BD Vacutainer, NJ) at the time of enrollment, at week 4, at the end of treatment (week 8 or week 12 depending on treatment regimen) and 12 weeks after therapy cessation (week 20-24 after HCV DAA therapy initiation). Plasma samples were stored at -20C. Plasma samples were measured for ATX, sCD14, IL-6, soluble CD163 (sCD163) (R&D Systems, Minneapolis, MN), and Mac2BP (Affymetrix-eBioscience, Vienna, Austria) by ELISA.

### Transient Elastography

Liver stiffness measurement was performed using transient elastography (TE) (FibroScan model 502, Echosens, France). Ten successful measurements were performed for each patient and only those obtained with a success rate of at least 60% and an interquartile range/median value (IQR/M) less than 30% were considered reliable. The results were expressed in kilopascals (kPa). Measurements were obtained at baseline before starting therapy (standard of care) and 6 months after completion of DAA HCV therapy (the latter measurement for standard of care or as part of this research protocol).

### Statistical Analysis

Statistical analyses were performed using SPSS for Windows v. 24.0 (IBM Corp, Armonk, New York). Associations between continuous variables were evaluated using Spearman rank correlation coefficient. Group comparisons were analyzed by Mann-Whitney U test. All tests of significance were 2-sided and *P* values of ≤ 0.05 were considered significant.

## RESULTS

Study participant characteristics are reflective of a Midwest VA patient population, with male predominance and genotype 1 HCV infection (Table 1). Prior to therapy, serum level of AST and APRI score positively correlated with sCD14, sCD163, and Mac2BP (Table 2, AST: r = 0.650, *P* = 0.016; r = 0.591, *P* = 0.012; r = 0.565, *P* = 0.028; APRI: r = 0.560, *P* = 0.049; r = 0.750, *P* = 0.001; r = 0.586, *P* = 0.022 respectively), while ALT positively correlated with Mac2BP (r = 0.705, *P* = 0.007). Albumin level negatively correlated with IL-6 and ATX level (r = -0.631, *P* = 0.012; and r = -0.494, *P* = 0.05; respectively). Platelet level negatively correlated with sCD163 (r = -0.584, *P* = 0.022). Levels of ATX and Mac2BP positively correlated with sCD14 and IL-6 before start of therapy (ATX: r = 0.490, *P* = 0.046; r = 0.556, *P* = 0.01; Mac2BP: r = 0.588, *P* = 0.017; r = 0.563, *P* = 0.040 respectively). No correlation was observed between TE score and HCV level or clinical parameters before initiation of IFN-free therapy (Table 2).

**Table 2:** Correlations between parameters before IFN-free DAA therapy

	AST	ALT	PLT	ALB	APRI	TE	HCV	ATX	sCD14	sCD163	Mac2BP	IL-6
**AST**		R = 0.881 *P* < 0.001			R = 0.936 *P* < 0.001				R = 0.650 *P* = 0.016	R = 0.591 *P* = 0.012	R = 0.565 *P* = 0.028	
**ALT**	R = 0.881 *P* < 0.001				R = 0.831 *P* < 0.001						R = 0.705 *P* = 0.007	
**ALB**								R = −0.494 *P* = 0.05				R = −0.631 *P* = 0.012
**PLT**					R = −584 *P* = 0.009					R = −0.584 *P* = 0.022		
**APRI**	R = 0.936 *P* < 0.001	R = 0.831 *P* < 0.001	R = −0.584 *P* = 0.009						R = 0.560 *P* = 0.049	R = 0.750 *P* = 0.001	R = 0.586 *P* = 0.022	
**TE**												
**HCV**												
**ATX**				R = −0.494 *P* = 0.05					R = 0.490 *P* = 0.046			R = 0.556 *P* = 0.01
**sCD14**	R = 0.650 *P* = 0.016				R = 0.560 *P* = 0.049			R = 0.490 *P* = 0.046			R = 0.588 *P* = 0.017	
**sCD163**	R = 0.591 *P* = 0.012		R = −0.584 *P* = 0.022		R = 0.750 *P* = 0.001							
**Mac2BP**	R = 0.565 *P* = 0.028	R = 0.705 *P* = 0.007			R = 0.586 *P* = 0.022				R = 0.588 *P* = 0.017			R = 0.563 *P* = 0.040
**IL-6**				R = −0.631 *P* = 0.012				R = 0.556 *P* = 0.01			R = 0.563 *P* = 0.040	

Correlations were determined between clinical parameters (AST, ALT, PLT, ALB, HCV, TE) and soluble markers of immune activation at baseline, before therapy, and those parameters which are significantly correlated (*P*<0.05) are shown along with correlation coefficient.

**Figure 1.**
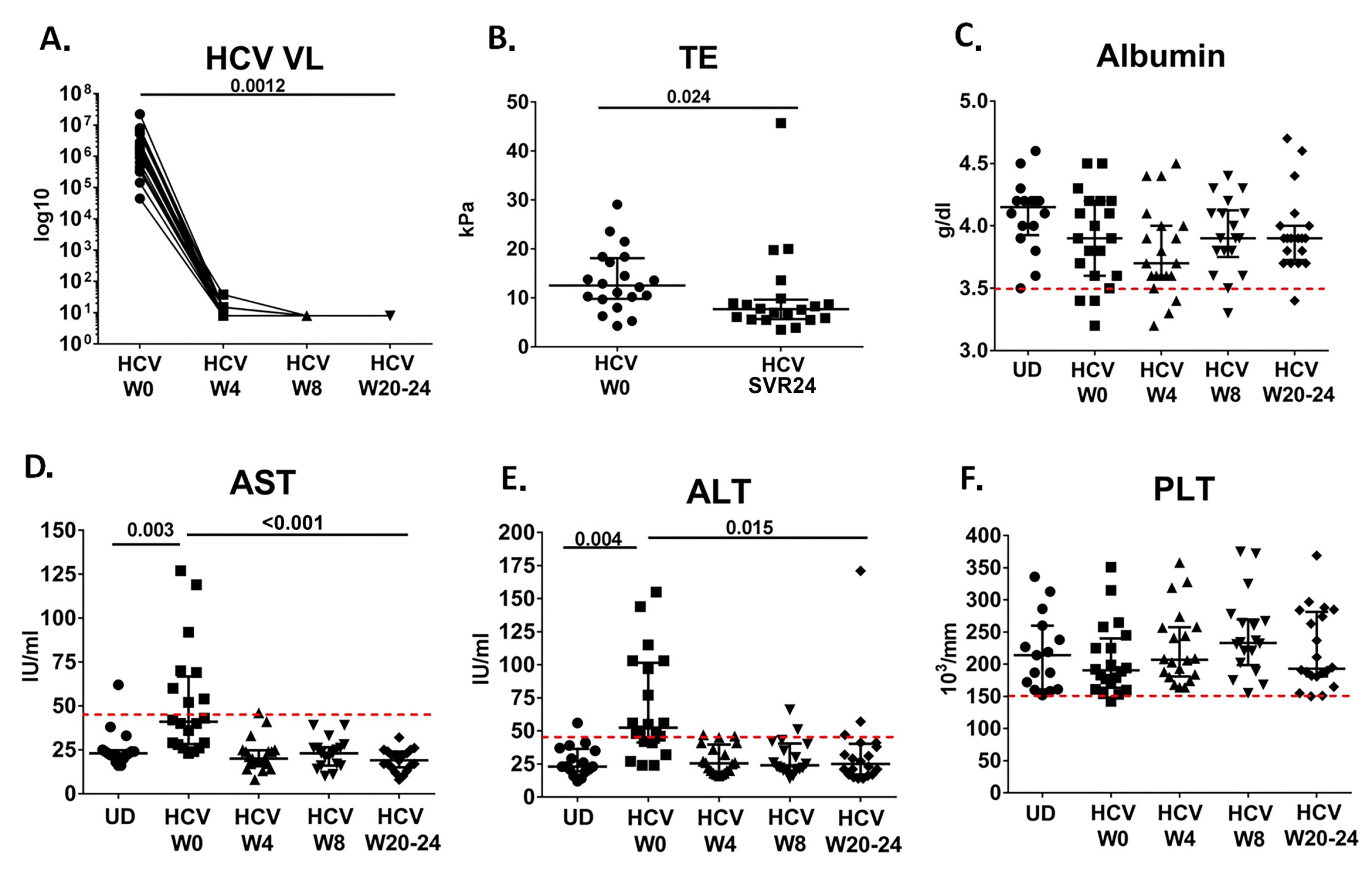
**Normalization of clinical parameters over the course of IFN-free DAA therapy.** A) Plasma HCV viral level is not detectable (lower limit of detection 15 IU/ml) at week 4 after starting DAA therapy. Log10 HCV viral level is shown over time after start of HCV DAA therapy at week 0 (w0). B) TE score (significantly decreases at week 24 after completion of DAA therapy SVR24 (*P* = 0.024). C) Albumin level over course of therapy in HCV-infected cohort as well as at 1 time point in uninfected donors (UD). Red dotted line indicates lower limit of normal. D) AST level over course of therapy in HCV-infected cohort (W0, W4, W8, and W20-24) as well as at 1 time point in uninfected donors (UD). Red dotted line indicates upper limit of normal. E) ALT level over course of therapy in HCV-infected cohort as well as at 1 time point in uninfected donors (UD). Red dotted line indicates upper limit of normal. F) PLT level over course of therapy in HCV-infected cohort as well as at 1 time point in uninfected donors. Red dotted line indicates lower limit of normal.

**Figure 2.**
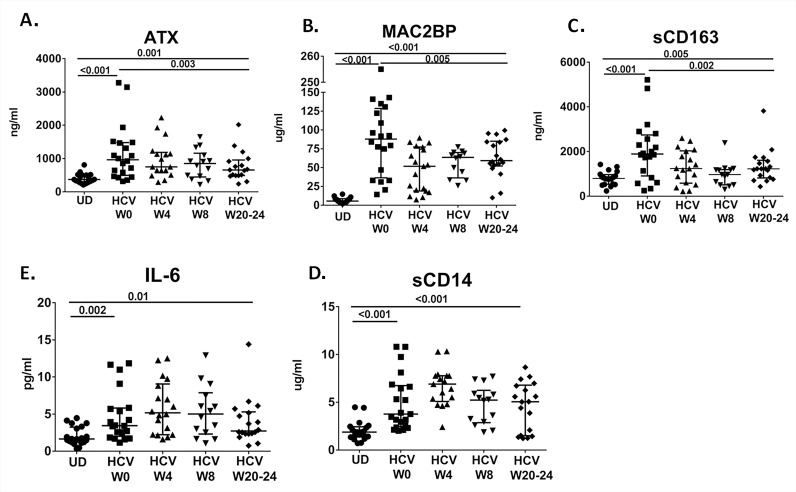
**Partial normalization of inflammatory parameters over the course of IFN-free DAA therapy.** Plasma levels of A) Autotaxin (ATX), B) Mac2BP, C) sCD163, D) IL-6 and E) sCD14 are shown over the course of IFN-free DAA therapy in HCV-infected participants (W0, W4, W8, and W20-24). Also shown are levels of each soluble factor for uninfected donors (UD).

During IFN-free DAA therapy we observed complete normalization of the AST and ALT level. HCV was not detectable at week 4 after the start of therapy in all participants (Figure 1), and all participants achieved SVR12 and SVR24. Platelet and albumin levels were mostly within normal range at baseline, and no significant change was seen over the course of therapy (Figure 1). TE score had decreased at 6 months following completion of therapy or SVR24 (*P* = 0.024, Figure 1). Additionally, we observed partial normalization of the levels of ATX, Mac2BP, and sCD163 (*P* = 0.003, *P* = 0.005, and *P* = 0.002, Figure 2); however, the reduction in levels was still different when compared to uninfected controls (*P* < 0.001 for each, Figure 2). No significant changes were observed in the levels of sCD14 or IL-6 during the 6 months following initiation of curative DAA IFN-free HCV therapy (Figure 2), although in a number of cases it appeared that sCD14 levels increased by week 4 of therapy. Only when evaluating sCD14 over the course of therapy in the subset of participants with higher TE scores (> median, 12.5 kPa) at baseline did we observe a significant decline in sCD14 level, comparing week 0 to week 20-24 (n = 10, *P* = 0.02).

**Figure 3.**
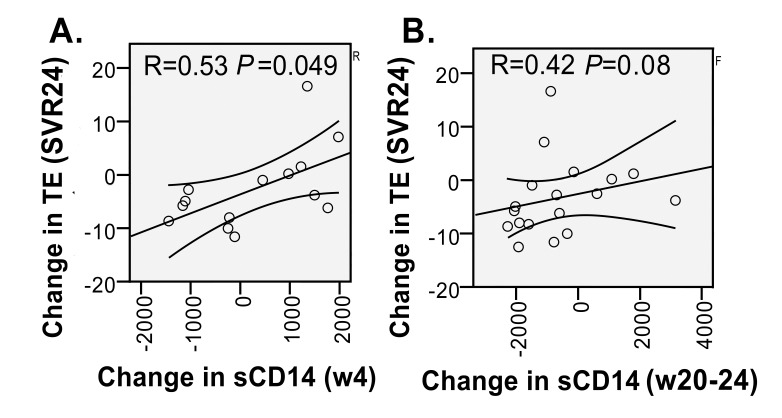
**Correlation between change in sCD14 and change in TE score over the course of IFN-free therapy.** A) Change in sCD14 (week 4 [w4]-baseline) vs Change in TE score (SVR24 or 24 weeks post therapy-baseline). B) Change in sCD14 (week 20-24 [w20-24]-baseline) vs Change in TE score (SVR24 or 24 weeks post therapy-baseline). Spearman rank correlation coefficient and *P* value are shown in each panel and 95% confidence intervals.

After initiation of IFN-free HCV therapy, change in AST level correlated with change in sCD163 and IL-6 levels (r = 0.703, *P* = 0.002; and r = 0.547, *P* = 0.002), and change in ALT correlated with change in IL-6 level (r = 0.529, *P* = 0.024). There also was a negative correlation between change in albumin and change in ATX and Mac2BP levels at week 4 (r = -0.629, *P* = 0.016; and r = -0.776, *P* = 0.001). Improvement in TE score was only correlated with decrease in the sCD14 at week 4, and nearly with decrease in sCD14 at week 20-24 after initiation of DAA HCV therapy (Figure 3).

## DISCUSSION

We observed correlations between AST/ALT and soluble markers of monocyte/Kupffer cell activation (sCD14, sCD163), as well as between serum albumin level (a marker of liver synthetic function and nutritional status) and plasma levels of IL-6 and ATX prior to IFN-free HCV therapy. During therapy, AST and ALT normalized, while ATX, sCD163, and Mac2BP levels partially normalized. IL-6 and sCD14 did not normalize. However, sCD14 change correlated with normalization of TE score. The latter provides support for a model where monocyte/Kupffer cell activation contributes to a reversible component of liver stiffness during chronic HCV infection. A limitation of this study is that this is a small sample set, and there were no corrections for multiple comparisons. However, our primary intent was to understand those soluble factors that were associated with TE score or change in TE score.

There is a growing literature regarding degree and rate of normalization of liver stiffness during DAA HCV therapy [12–15]. In patients who achieve SVR, there is a significant decrease in TE 12 months after therapy and greater decreases in patients treated with DAA compared to IFN-based therapy [12]. The decrease in the levels of AST and ALT and the increase in platelet levels and albumin has been shown in both DAA and IFN-based therapies for HCV [14, 15, 31, 32]. In patients with HCV mono-infection, treated with either DAA or IFN-based therapy, there is a significant association between the decrease in AST and ALT and percentage decline of TE [15]. Additionally, decreased ALT, decreased hyaluronic acid level, and increased duration of treatment have been associated with a decrease in fibrotic stage measured by TE [14]. In HCV/HIV co-infected patients Charpentier *et al* showed that AST, ALT, and platelet levels are associated with the stage of liver fibrosis, and sCD14 was positively associated with F3-F4 liver fibrosis [33]. In contrast, Medrano *et al* showed no correlation between sCD14 and TE score in HCV-HIV co-infected patients, but patients with higher TE scores had higher proportions of CD8+CD38+ T cells and higher plasma levels of IL-8, IL-6, IP-10, and LPS [34]. Our work here provides a first look at the relationship between soluble markers of immune activation and TE score during HCV mono-infection and how the changes in these parameters over the course of therapy relate to each other.

A number of studies have examined the rate of normalization of parameters of immune activation and inflammation over the course of HCV DAA therapy [22, 29, 30, 35]. We have previously demonstrated that ATX levels were associated with IL-6, sCD14, sCD163, Mac2BP, and Lysophosphatidic acid (LPA) levels in HCV-infection, and that ATX, LPA, and sCD14 levels normalized, while CD163 and Mac2BP levels partially normalized within 6 months of starting IFN-free DAA HCV therapy [22]. This is further supported and extended by the findings of Yamazaki *et al*, where a significant decline in ATX level was observed in participants who achieved SVR after IFN-free DAA therapy compared to those who did not [36]. Additionally the incomplete normalization of soluble inflammatory markers over the course of IFN-free therapy found in our study aligns with findings of Mascia *et al*, who evaluated CXCL10 and sCD163 levels over the course of IFN-free therapy [30]. One difference between our prior study and the present is that sCD14 normalized or partially normalized in low and high APRI subgroups in our prior data set, while here sCD14 levels did not significantly change in the group as a whole, although there was a trend in this direction (*P* = 0.058). These differences between studies may be attributable to different patient population characteristics. Here, participants were selected based on transient elastography longitudinal analysis, where participants either had TE repeated for clinical purposes or research purposes. Those that had TE repeated for clinical purposes generally had higher scores at baseline than our overall treated patient population (median 12.5 here compared with 8.5 for our overall HCV treatment cohort); these scores may be higher than those of our prior published cohort where TE scores were available for only a minority of participants (median 11.3).

Consistent with this possibility, the sCD14 levels at baseline are higher in the present data set. In fact, when analyzing sCD14 over the course of therapy in those participants with TE score >12.5 at baseline we observe a significant decline in sCD14 level comparing week 0 to week 20-24 (n = 10, *P* = 0.02). It appears overall that sCD14 changes with therapy in a subset of individuals, with variation in degree and timing of change that is associated with TE score at baseline and TE change. The latter supports a model where monocyte/Kupffer cell activation contributes to the inflammatory state resulting in a component of liver stiffness that, on initiation of DAA therapy, partially normalizes. The concept of a reversible measure of monocyte/Kupffer cell activation in fibrogenesis is not new. Lidofsky *et al* have shown that sCD163 and liver macrophage CD163 are associated with liver fibrogenesis, but not total liver fibrosis stage in the setting of HCV/HIV infection, and nearly the case in the setting of HCV infection as well [37]. This places macrophages in the center of the fibrogenic process in both HCV/HIV infection and HCV mono-infection. Whether prolonged elevation of sCD14 or sCD163 levels after IFN-free DAA therapy predict future liver-related events is not known, and whether rates of sCD14 or sCD163 normalization reflect a mix of primary and secondary drivers of monocyte/macrophage/Kupffer cell activation is unclear. Certainly, potential secondary drivers, such as LPS and LPA may be the consequence of liver damage itself as opposed to active HCV infection and have been proposed to contribute to immune dysfunction and continued liver damage [22, 38, 39]. How this relates to overall liver health, immune restoration, and/or improvement in risk for HCC is yet to be determined.

## References

[1] Liang TJ, Rehermann B, Seeff LB, Hoofnagle JH Pathogenesis, natural history, treatment, and prevention of hepatitis C. Ann Intern Med. 2000;132(4):296–305. PubMed PMID: 10681285. 10681285 10.7326/0003-4819-132-4-200002150-00008

[2] Ansaldi F, Orsi A, Sticchi L, Bruzzone B, Icardi G Hepatitis C virus in the new era: perspectives in epidemiology, prevention, diagnostics and predictors of response to therapy. World J Gastroenterol. 2014;20(29):9633–52. PubMed PMID: 25110404. Pubmed Central PMCID: PMC4123355. doi: 10.3748/wjg.v20.i29.9633 25110404 PMC4123355

[3] EASL-EORTC clinical practice guidelines: management of hepatocellular carcinoma. J Hepatol. 2012;56(4):908–43. PubMed PMID: 22424438. doi: 10.1016/j.jhep.2011.12.001 22424438

[4] Fried MW, Shiffman ML, Reddy KR, Smith C, Marinos G, Goncales FL Jr., Haussinger D, Diago M, Carosi G, Dhumeaux D, Craxi A, Lin A, Hoffman J, Yu J Peginterferon alfa-2a plus ribavirin for chronic hepatitis C virus infection. N Engl J Med. 2002;347(13):975–82. PubMed PMID: 12324553. doi: 10.1056/NEJMoa020047 12324553

[5] Hadziyannis SJ, Sette H Jr., Morgan TR, Balan V, Diago M, Marcellin P, Ramadori G, Bodenheimer H Jr., Bernstein D, Rizzetto M, Zeuzem S, Pockros PJ, Lin A, Ackrill AM, Group PIS Peginterferon-alpha2a and ribavirin combination therapy in chronic hepatitis C: a randomized study of treatment duration and ribavirin dose. Ann Intern Med. 2004;140(5):346–55. PubMed PMID: 14996676. 14996676 10.7326/0003-4819-140-5-200403020-00010

[6] Lam BP, Jeffers T, Younoszai Z, Fazel Y, Younossi ZM The changing landscape of hepatitis C virus therapy: focus on interferon-free treatment. Therap Adv Gastroenterol. 2015;8(5):298–312. PubMed PMID: 26327920. Pubmed Central PMCID: PMC4530432. doi: 10.1177/1756283X15587481 PMC4530432 26327920

[7] Simmons B, Saleem J, Heath K, Cooke GS, Hill A Long-Term Treatment Outcomes of Patients Infected With Hepatitis C Virus: A Systematic Review and Meta-analysis of the Survival Benefit of Achieving a Sustained Virological Response. Clin Infect Dis. 2015;61(5):730–40. PubMed PMID: 25987643. Pubmed Central PMCID: PMC4530725. doi: 10.1093/cid/civ396 25987643 PMC4530725

[8] Backus LI, Belperio PS, Shahoumian TA, Mole LA Direct-Acting Antiviral Sustained Virologic Response: Impact on Mortality in Patients without Advanced Liver Disease. Hepatology. 2018 PubMed PMID: 29377196. doi: 10.1002/hep.29811 29377196

[9] Grandhe S, Frenette CT Occurrence and Recurrence of Hepatocellular Carcinoma After Successful Direct-Acting Antiviral Therapy for Patients With Chronic Hepatitis C Virus Infection. Gastroenterol Hepatol (N Y). 2017;13(7):421–5. PubMed PMID: 28867970. Pubmed Central PMCID: PMC5572972. 28867970 PMC5572972

[10] Sandrin L, Fourquet B, Hasquenoph JM, Yon S, Fournier C, Mal F, Christidis C, Ziol M, Poulet B, Kazemi F, Beaugrand M, Palau R Transient elastography: a new noninvasive method for assessment of hepatic fibrosis. Ultrasound Med Biol. 2003;29(12):1705–13. PubMed PMID: 14698338. 14698338 10.1016/j.ultrasmedbio.2003.07.001

[11] Talwalkar JA, Kurtz DM, Schoenleber SJ, West CP, Montori VM Ultrasound-based transient elastography for the detection of hepatic fibrosis: systematic review and meta-analysis. Clin Gastroenterol Hepatol. 2007;5(10):1214–20. PubMed PMID: 17916549. doi: 10.1016/j.cgh.2007.07.020 17916549

[12] Singh S, Facciorusso A, Loomba R, Falck-Ytter YT Magnitude and Kinetics of Decrease in Liver Stiffness After Antiviral Therapy in Patients With Chronic Hepatitis C: A Systematic Review and Meta-analysis. Clin Gastroenterol Hepatol. 2018;16(1):27-38 e4. PubMed PMID: 28479504. Pubmed Central PMCID: PMC5671365. doi: 10.1016/j.cgh.2017.04.038 28479504 PMC5671365

[13] Facciorusso A, Del Prete V, Turco A, Buccino RV, Nacchiero MC, Muscatiello N Long-term liver stiffness assessment in hepatitis C virus patients undergoing antiviral therapy: Results from a 5-year cohort study. J Gastroenterol Hepatol. 2017 PubMed PMID: 28976021. doi: 10.1111/jgh.14008 28976021

[14] Arima Y, Kawabe N, Hashimoto S, Harata M, Nitta Y, Murao M, Nakano T, Shimazaki H, Kobayashi K, Ichino N, Osakabe K, Nishikawa T, Okumura A, Ishikawa T, Yoshioka K Reduction of liver stiffness by interferon treatment in the patients with chronic hepatitis C. Hepatol Res. 2010;40(4):383–92. PubMed PMID: 20236358. doi: 10.1111/j.1872-034X.2009.00618.x 20236358

[15] Chekuri S, Nickerson J, Bichoupan K, Sefcik R, Doobay K, Chang S, DelBello D, Harty A, Dieterich DT, Perumalswami PV, Branch AD Liver Stiffness Decreases Rapidly in Response to Successful Hepatitis C Treatment and Then Plateaus. PLoS One. 2016;11(7):e0159413 PubMed PMID: 27442255. Pubmed Central PMCID: PMC4956253. doi: 10.1371/journal.pone.0159413 27442255 PMC4956253

[16] Negash AA, Ramos HJ, Crochet N, Lau DT, Doehle B, Papic N, Delker DA, Jo J, Bertoletti A, Hagedorn CH, Gale M Jr IL-1beta production through the NLRP3 inflammasome by hepatic macrophages links hepatitis C virus infection with liver inflammation and disease. PLoS Pathog. 2013;9(4):e1003330 PubMed PMID: 23633957. Pubmed Central PMCID: PMC3635973. doi: 10.1371/journal.ppat.1003330 23633957 PMC3635973

[17] Szabo G, Mandrekar P, Dolganiuc A Innate immune response and hepatic inflammation. Semin Liver Dis. 2007;27(4):339–50. PubMed PMID: 17979071. doi: 10.1055/s-2007-991511 17979071

[18] Ahlenstiel G, Titerence RH, Koh C, Edlich B, Feld JJ, Rotman Y, Ghany MG, Hoofnagle JH, Liang TJ, Heller T, Rehermann B Natural killer cells are polarized toward cytotoxicity in chronic hepatitis C in an interferon-alfa-dependent manner. Gastroenterology. 2010;138(1):325-35 e1-2. PubMed PMID: 19747917. Pubmed Central PMCID: PMC2862622. doi: 10.1053/j.gastro.2009.08.066 19747917 PMC2862622

[19] Bility MT, Nio K, Li F, McGivern DR, Lemon SM, Feeney ER, Chung RT, Su L Chronic hepatitis C infection-induced liver fibrogenesis is associated with M2 macrophage activation. Sci Rep. 2016;6:39520 PubMed PMID: 28000758. Pubmed Central PMCID: PMC5175173. doi: 10.1038/srep39520 28000758 PMC5175173

[20] Sandler NG, Koh C, Roque A, Eccleston JL, Siegel RB, Demino M, Kleiner DE, Deeks SG, Liang TJ, Heller T, Douek DC Host response to translocated microbial products predicts outcomes of patients with HBV or HCV infection. Gastroenterology. 2011;141(4):1220-30, 30 e1-3. PubMed PMID: 21726511. Pubmed Central PMCID: PMC3186837. doi: 10.1053/j.gastro.2011.06.063 21726511 PMC3186837

[21] Kazankov K, Barrera F, Moller HJ, Bibby BM, Vilstrup H, George J, Gronbaek H Soluble CD163, a macrophage activation marker, is independently associated with fibrosis in patients with chronic viral hepatitis B and C. Hepatology. 2014;60(2):521–30. PubMed PMID: 24623375. doi: 10.1002/hep.27129 24623375

[22] Kostadinova L, Shive CL, Judge C, Zebrowski E, Compan A, Rife K, Hirsch A, Falck-Ytter Y, Schlatzer DM, Li X, Chance MR, Rodriguez B, Popkin DL, Anthony DD During Hepatitis C Virus (HCV) Infection and HCV-HIV Coinfection, an Elevated Plasma Level of Autotaxin Is Associated With Lysophosphatidic Acid and Markers of Immune Activation That Normalize During Interferon-Free HCV Therapy. J Infect Dis. 2016;214(9):1438–48. PubMed PMID: 27540113. doi: 10.1093/infdis/jiw372 27540113 PMC6281372

[23] Hiraoka A, Horiike N, Akbar SM, Michitaka K, Matsuyama T, Onji M Expression of CD163 in the liver of patients with viral hepatitis. Pathol Res Pract. 2005;201(5):379–84. PubMed PMID: 16047947. 16047947 10.1016/j.prp.2004.10.006

[24] Sandahl TD, Gronbaek H, Moller HJ, Stoy S, Thomsen KL, Dige AK, Agnholt J, Hamilton-Dutoit S, Thiel S, Vilstrup H Hepatic macrophage activation and the LPS pathway in patients with alcoholic hepatitis: a prospective cohort study. Am J Gastroenterol. 2014;109(11):1749–56. PubMed PMID: 25155228. doi: 10.1038/ajg.2014.262 25155228

[25] Gronbaek H, Sandahl TD, Mortensen C, Vilstrup H, Moller HJ, Moller S Soluble CD163, a marker of Kupffer cell activation, is related to portal hypertension in patients with liver cirrhosis. Aliment Pharmacol Ther. 2012;36(2):173–80. PubMed PMID: 22591184. doi: 10.1111/j.1365-2036.2012.05134.x 22591184

[26] Kitchens RL, Thompson PA Modulatory effects of sCD14 and LBP on LPS-host cell interactions. J Endotoxin Res. 2005;11(4):225–9. PubMed PMID: 16176659. doi: 10.1179/096805105X46565 16176659

[27] Wright SD, Ramos RA, Tobias PS, Ulevitch RJ, Mathison JC CD14, a receptor for complexes of lipopolysaccharide (LPS) and LPS binding protein. Science. 1990;249(4975):1431–3. PubMed PMID: 1698311. 1698311 10.1126/science.1698311

[28] Shive CL, Jiang W, Anthony DD, Lederman MM Soluble CD14 is a nonspecific marker of monocyte activation. AIDS. 2015;29(10):1263–5. PubMed PMID: 26035325. Pubmed Central PMCID: PMC4452959. doi: 10.1097/QAD.0000000000000735 26035325 PMC4452959

[29] Yamazaki T, Joshita S, Umemura T, Usami Y, Sugiura A, Fujimori N, Shibata S, Ichikawa Y, Komatsu M, Matsumoto A, Igarashi K, Tanaka E Association of Serum Autotaxin Levels with Liver Fibrosis in Patients with Chronic Hepatitis C. Sci Rep. 2017;7:46705 PubMed PMID: 28425454. Pubmed Central PMCID: PMC5397977. doi: 10.1038/srep46705 28425454 PMC5397977

[30] Mascia C, Vita S, Zuccala P, Marocco R, Tieghi T, Savinelli S, Rossi R, Iannetta M, Pozzetto I, Furlan C, Mengoni F, Mastroianni CM, Vullo V, Lichtner M Changes in inflammatory biomarkers in HCV-infected patients undergoing direct acting antiviral-containing regimens with or without interferon. PLoS One. 2017;12(6):e0179400 PubMed PMID: 28636655. Pubmed Central PMCID: PMC5499435. doi: 10.1371/journal.pone.0179400 28636655 PMC5499435

[31] Deterding K, Honer Zu Siederdissen C, Port K, Solbach P, Sollik L, Kirschner J, Mix C, Cornberg J, Worzala D, Mix H, Manns MP, Cornberg M, Wedemeyer H Improvement of liver function parameters in advanced HCV-associated liver cirrhosis by IFN-free antiviral therapies. Aliment Pharmacol Ther. 2015;42(7):889–901. PubMed PMID: 26250762. doi: 10.1111/apt.13343 26250762

[32] George SL, Bacon BR, Brunt EM, Mihindukulasuriya KL, Hoffmann J, Di Bisceglie AM Clinical, virologic, histologic, and biochemical outcomes after successful HCV therapy: a 5-year follow-up of 150 patients. Hepatology. 2009;49(3):729–38. PubMed PMID: 19072828. Pubmed Central PMCID: PMC2731713. doi: 10.1002/hep.22694 19072828 PMC2731713

[33] Charpentier C, Champenois K, Gervais A, Landman R, Joly V, Le Gac S, Larrouy L, Damond F, Brun-Vezinet F, Descamps D, Yazdanpanah Y Predictive value of liver enzymes and inflammatory biomarkers for the severity of liver fibrosis stage in HIV/HCV co-infected patients. PLoS One. 2013;8(3):e59205 PubMed PMID: 23527135. Pubmed Central PMCID: PMC3602202. doi: 10.1371/journal.pone.0059205 23527135 PMC3602202

[34] Medrano LM, Garcia-Broncano P, Berenguer J, Gonzalez-Garcia J, Jimenez-Sousa MA, Guardiola JM, Crespo M, Quereda C, Sanz J, Canorea I, Carrero A, Hontanon V, Munoz-Fernandez MA, Resino S, Group GbS Elevated liver stiffness is linked to increased biomarkers of inflammation and immune activation in HIV/hepatitis C virus-coinfected patients. AIDS. 2018;32(9):1095–105. PubMed PMID: 29438197. doi: 10.1097/QAD.0000000000001787 29438197

[35] Malone DF, Falconer K, Weiland O, Sandberg JK The dynamic relationship between innate immune biomarkers and interferon-based treatment effects and outcome in hepatitis C virus infection is altered by telaprevir. PLoS One. 2014;9(8):e105665 PubMed PMID: 25166593. Pubmed Central PMCID: PMC4148339. doi: 10.1371/journal.pone.0105665 25166593 PMC4148339

[36] Yamazaki T, Joshita S, Umemura T, Usami Y, Sugiura A, Fujimori N, Kimura T, Matsumoto A, Igarashi K, Ota M, Tanaka E Changes in serum levels of autotaxin with direct-acting antiviral therapy in patients with chronic hepatitis C. PLoS One. 2018;13(4):e0195632 PubMed PMID: 29617443. Pubmed Central PMCID: 5884565. doi: 10.1371/journal.pone.0195632 29617443 PMC5884565

[37] Lidofsky A, Holmes JA, Feeney ER, Kruger AJ, Salloum S, Zheng H, Seguin IS, Altinbas A, Masia R, Corey KE, Gustafson JL, Schaefer EA, Hunt PW, Deeks S, Somsouk M, Chew KW, Chung RT, Alatrakchi N Macrophage Activation Marker Soluble CD163 is a Dynamic Marker of Liver Fibrogenesis in HIV/HCV Coinfection. J Infect Dis. 2018 PubMed PMID: 29868909. doi: 10.1093/infdis/jiy331 PMC6151081 29868909

[38] Balagopal A, Philp FH, Astemborski J, Block TM, Mehta A, Long R, Kirk GD, Mehta SH, Cox AL, Thomas DL, Ray SC Human immunodeficiency virus-related microbial translocation and progression of hepatitis C. Gastroenterology. 2008;135(1):226–33. PubMed PMID: 18457674. Pubmed Central PMCID: PMC2644903. doi: 10.1053/j.gastro.2008.03.022 18457674 PMC2644903

[39] McGovern BH, Golan Y, Lopez M, Pratt D, Lawton A, Moore G, Epstein M, Knox TA The impact of cirrhosis on CD4+ T cell counts in HIV-seronegative patients. Clin Infect Dis. 2007;44(3):431–7. PubMed PMID: 17205454. doi: 10.1086/509580 17205454

